# Biochemical analysis of miR-217 and miR-532 in patients with fibromyalgia

**DOI:** 10.1186/s40001-025-02330-y

**Published:** 2025-02-07

**Authors:** Shymaa E. Ayoub, Azza M. Ahmed, Mostafa Y. Abdelwahed, Abeer A. Khalefa, Aeshah A. Awaji, Samer S. Zekry, Enas G. Ibrahim, Hana M. Eid, Shimaa M. Elasmer, Reham Fares

**Affiliations:** 1 Medical Biochemistry and Molecular Biology Department, Faculty of Medicine, Fayoum University, Al Fayoum, Egypt; 2 Rheumatology Department, Faculty of Medicine, Fayoum University, Fayoum, Egypt; 3 Department of Physiology, Faculty of Medicine, Fayoum University, Fayoum, Egypt; 4 Department of Physiology, Faculty of Medicine, Zagazig University, Zagazig, Egypt; 5 Department of Biology, Faculty of Science, University College of Taymaa, University of Tabuk, 71491 Tabuk, Saudi Arabia; 6 Department of Internal Medicine, Faculty of Medicine, Fayoum University, Fayoum, Egypt; 7 Department of Medical Microbiology and Immunology, Faculty of Medicine, Fayoum University, Fayoum, Egypt; 8 Department of Clinical and Chemical Pathology, Faculty of Medicine, Fayoum University, Fayoum, Egypt

**Keywords:** Fibromyalgia, MiR**-**217, MiR-532

## Abstract

About 5% of the population suffers from fibromyalgia (FM), a chronic multi-symptom pain illness whose pathophysiology is still unknown. We aimed to be the first to investigate the possible association of sera levels of miR**-**217 and miR-532 in patients with fibromyalgia and correlate their expression levels to different clinical and biochemical disease criteria. This study included 80 participants who splitted into two groups: 40 fibromyalgia sufferers (12 male and 28 female), and 40 healthy volunteers (10 male and 30 female) who served as the control group. Venous blood samples were collected from all subjects. The miR**-**217 and miR-532 serum expressions were detected using quantitative real-time PCR (qRT-PCR). According to our data, the fold changes of miR**-**217 and miR-532 in fibromyalgia patients were significantly lower than in controls, for miR**-**217 (median = 0.1359, IQR: 0.038–0.287, P < 0.001) and miR-532 [median = 0.2199, IQR: (0.114–0.421), *P* < 0.001]. In addition, there was a significant negative relationship between Aspartate transaminase (AST) and both miR-217 and miR**-**532 (*r* = − 0.480, *P* = *P* < 0.001 *r* = − 0.462, *P* = *P* < 0.001), respectively. Serum miR**-**217 and miR-532 could serve as potential diagnostic biomarkers for fibromyalgia.

## Introduction

Fibromyalgia (FM) is a chronic condition characterized by widespread, persistent pain (nociplastic pain). It is also linked to fatigue, sleep, cognitive problems, and somatic complaints [1]. Treatment failures are common, and their clinical, physical, social, and psychological symptoms are diverse [2]. According to estimates, the general population has a 1.78% prevalence of FM, with a predominance of women [3]. The American College of Rheumatology's recommendations [4] are followed for the diagnosis of FM, and international guidelines for managing the condition first suggest non-pharmacological approaches (exercise), followed by drug treatment for pain and comorbidities (although quality of life is frequently compromised). Although the primary mechanism thought to be responsible for fibromyalgia is central sensitization, numerous other genetic, immunological, and hormonal variables may also be significant contributors [5].

Many FM patients have missed or delayed diagnoses, which not only compromises treatment outcomes but also raises the cost of healthcare.

Even though several biomarkers and other prognostic indicators for FM have been proposed [6]. No particular biomarker has been found yet. The identification of indicators that are regularly linked to this pathology would help clinicians diagnose FM more accurately, track the disease's progression, evaluate the effectiveness of treatment options, and even create preventive initiatives. Although fibromyalgia was once thought to be a non-inflammatory ailment [7, 8], it is now known that patients with the condition have activated numerous pro-inflammatory pathways and mediators [9, 10]. Numerous clinical and experimental studies demonstrated the critical role that inflammation plays in fibromyalgia. [11].

MicroRNAs (miRNAs) are among the most promising classes of molecules that have the potential to be useful biomarkers. MiRNAs are a subclass of short non-coding RNAs that control post-transcriptional levels of gene expression and are crucial for physiological and developmental processes [12]. Furthermore, by sequencing blood samples from patients with FM, several researchers have found many genes and miRNA molecules that may be utilized for an early diagnosis of FM [13].

MiR-217, which is found on human chromosome 2p16.1, is crucial for fibrosis and inflammation [14]. MiR-217-5p is greatly upregulated during the course of endothelial cell senescence and dysfunction in cardiovascular disease (CVD) and has a strong correlation with aging [15, 16]**.** Also linked to the apoptosis of podocyte in diabetic nephropathy [17] and macrophage apoptosis in atherosclerosis [18]. Furthermore, MiR-217 has been studied in several cancers, including acute myeloid leukemia [19] and liver cancer [20]. In addition, the inhibition of miR-217 was associated with improvement of manifestations in Parkinson’s disease [21].

MIR-532 (MicroRNA-532) is located on (Xp11.23). Numerous physiological functions have been linked to miR-532 including cell migration, proliferation, metabolism, and apoptosis, [22, 23]**.** By controlling cell division, migration, colony formation, and apoptosis, miR-532 dysregulation can function as a tumor suppressor [24, 25] or oncogene [23, 26] and contribute to the growth of tumors.

In this study, we revealed the expression profile of miR-217 and miR-532in cases with fibromyalgia and explored their association with each other and with the clinicopathological manifestations of patients.

## Subjects and methods

We studied 80 participants in total; 40 were healthy individuals and 40 were fibromyalgia patients who were recruited from the Rheumatology and Internal Medicine departments, in Fayoum University Hospital, Egypt.

### lnclusion criteria

- Fibromyalgia diagnosis for at least 1 year.
- Age between 18 and 65 years.
- No pregnancy and breastfeeding.
- Cognitive integrity.
- Formal consent to study participation: before collecting samples, all participants gave their informed agreement, the Ethical Committee of the Faculty of Medicine, Fayoum University approved the protocol for this investigation (R598). This study is consistent with the Declaration of Helsinki.

### Exclusion criteria

- Expectant or nursing mothers.
- Cancer.
- Autoimmune illness.
- Diabetes and hypertension.
- Musculoskeletal inflammatory conditions.
- Severe heart diseases.
- Chronic abuse of alcohol.
- Neuropsychiatric disorder.

## Methods

### Serum preparation

Five milliliters of venous blood were obtained from both control and fibromyalgia patients. The samples were placed in serum separator tubes, where they were clotted for 15 min before being centrifuged for 10 min at 4000 revolutions per minute. Liver and kidney functions were done for both cases and control, for patients vitamin D, TSH, ESR CRP were done.

### Serum total RNA extraction including miRNAs

RNAs extraction from sera was obtained by (Qiagen, Valenica, CA, USA) extraction kits in accordance with the manufacturer protocol, The eluted RNA was split into two fractions: the larger component was held at −80°C until further analytical stages, and For the NanoDrop spectrophotometric RNA quantification and purity evaluation, the smaller fraction (5 µL) was utilized.

### MiRNAs reverse transcription into complementary DNA

For this step, Qiagen's miScript II RT Kit and the RNA reverse transcription (RT) technique into complementary DNA (cDNA) were utilized in consistence with the manufacturer's directions.

### Quantification of mature miRNAs using qRT-PCR

Utilizing the miScript SYBR Green PCR Kit (Qiagen, Germany), the cDNA templates underwent quantitative RT-PCR amplification. Each reaction had a total volume of 25 μL and was conducted with the particular miRNA-217 primer (Cat. No. YP00204010), miRNA-532 primer (Cat. No. YP00204003) and the housekeeping gene miR-16 (Cat. No. YP00205702), which serves as an endogenous control for relative quantification and data normalization. For both, the qRT-PCR was set up with the following cycle parameters: 95 °C was used for the first 15 min of the activation phase. Cycling denaturation was then done at 94 °C for 15 s, followed by annealing at 55 °C for 30 s, then 30 s at 70 °C for extension. For forty cycles, these processes were repeated. The 2^-ΔΔCt^ method was utilized for relative RNAs expression determination [27].

### Statistical analysis

GraphPad Prism 6.01, Systat SigmaPlot 12.5, and the Statistical Package for Social Sciences (SPSS) 22 were used for data analysis. The data were displayed as mean ± standard deviation (SD), number, percentage, and median (interquartile range, or IQR). When comparing variables between different groups, the Mann–Whitney *U* test was utilized if the variables were not normally distributed, and the independent *t* test was employed if they were. Numbers and percentages were employed to present the qualitative data, and the chi-square (*χ*^2^) test was applied to determine significance. The degree of association between various miRNAs and the clinicopathological data of the patients was ascertained by computing the correlation coefficient (r) using Pearson correlation. *P* values less than 0.05 were taken into account for determining the significance of (r).

## Results

### Assessment of the research groups' clinical and demographic characteristics

In this study, 40 participants with fibromyalgia were included as cases, and another 40 participants acted as controls. While the control group’s average age was 35 ± 8 years, the patients' average age was 37 ± 9 years. Between cases and controls, there was no discernible difference in age or sex. As regards serum levels of urea, creatinine, ALT and AST, There was a significant difference in AST level between the patients and control groups (*P* < 0.001), although we are unsure if this difference has any clinical implications (Table 1). Clinical characteristics of the case group regarding disease duration, widespread pain index, symptoms severity scale and polysymptomatic distress scale are demonstrated in Table 2.

**Table 1:** Demographic and biochemical characteristics of the study groups

	**Cases** **(*N* = 40)**		**Control** **(*N* = 40)**		***P* value**
	**Mean ± SD**	***N* (%)**	**Mean ± SD**	***N* (%)**	
Age	37 ± 9		35 ± 8		0.53
Sex					
Male		12(30%)		10 (25%)	0.62
Female		28(70%)		30(70%)	
Urea	28 ± 10		25 ± 11		0.12
Creatinine	0.82 ± 0.34		0.69 ± 0.22		0.05
AST IU/L	29 ± 5		22 ± 7		< 0.001
ALT IU/L	27 ± 4		26 ± 5		0.26
vit D ng/ml	28.8 ± 4.9				
TSH mIU/l	2.5 ± 9				
ESR mm/hr	10 ± 4				
CRP mg/dl	0.5 ± 0.2				

AST,aspartate transaminase; ALT, alanine transaminase; vit D, vitamin D; TSH,Thyroid stimulating hormone; ESR., Erythrocyte sedimentation Rate; CRP, C-reactive protein

Data are represented as mean ± standard deviation or n (%). ^*^ Significant at *P* < 0.05

**Table 2:** Clinical characteristics of the case group

	Mean ± SD	Median (IQR)
Disease duration	5.3 ± 4.4	4.0(2–6)
Widespread pain index	12 ± 4	12(10–14)
Symptoms severity scale	8 ± 3	9(6–11)
Polysymptomatic distress scale	21 ± 5	20(17–24)

### Evaluation of the fold change of miR-217 and miR-532 between the research groups

The fold changes of both miR**-**217 and miR-532 were significantly down-regulated in fibromyalgia patients as regards Median** ± **IQR of miR-217 [0.14 (0.04–0.29), *P* < 0.001], while miR-532 [0.22 (0.11–0.42), *P* < 0.001] (Table 3).

**Table 3:** Relative expression levels of serum miR-217and miR-532

	**Cases** **(*N* = 40)**	**Control** **(*N* = 40)**	***P*-value**^**#**^
MiR-217; Median ± IQR	0.14 (0.04–0.29)	1 ± 0	< 0.0001
MiR-532; Median ± IQR	0.22 (0.11–0.42)	1 ± 0	< 0.0001

Fold change values show target genes expressions compared to controls, as determined using 2^−ΔΔCt^. Levels of control fold change are equal to 1

### Correlation of the serum expression levels of miR-217 and miR-532 with clinicopathological data of the patients

Significant negative correlations were shown (*r* = − 0.48, *P* = 0.001) between AST and MiR-217 and between AST and MiR-532 (*r* = − 0.46, *P* = 0.001). However, it is unclear if these correlations have any clinical significance (Table 4).

**Table 4:** Spearman correlation of miR-217and miR-532 with study parameters in case group

	**MiR 217**	**MiR 532**
MiR 217		
r	1.00	0.89
P		0.00
MiR 532		
r	0.89	1.00
P	0.00	
Age		
r	− 0.03	− 0.07
P	0.82	0.50
Disease duration		
r	0.06	0.024
P	0.71	0.89
Widespread pain index		
r	0.08	0.29
P	0.65	0.07
Symptoms severity scale		
r	0.03	0.16
P	0.87	0.33
Polysymptomatic distress scale		
r	0.02	0.31
P	0.91	0.052
Urea		
r	− 0.18	− 0.19
P	0.17	0.09
N	80	80
Creatinine		
r	− 0.22	− 0.17
P	0.05	0.14
N	80	80
AST		
r	− 0.48	− 0.46
P	0.00	0.00
ALT		
r	− 0.10	− 0.13
P	0.40	0.26
N	80	80
Vit D ng/ml		
r	− 0.16	0.06
P	0.31	0.70
N	40	40
TSH mIU/l		
r	0.19	− 0.00
P	0.25	0.99
N	40	40
ESR mm/hr		
r	0.31	− 0.06
P	0.05	0.70
CRP mg/dl		
r	− 0.26	0.13
P	0.11	0.42

AST, aspartate transaminase; ALT, alanine transaminase; vit D, vitamin D; TSH, Thyroid stimulating hormone; ESR., Erythrocyte sedimentation Rate; CRP, C-reactive protein

## Discussion

Fibromyalgia (FM) is one of the most controversial medical disorders; it still has many unanswered questions from the pathogenetic, therapeutic, and diagnostic domains. Up until now, a clinician's judgment has been the primary gold standard for diagnosing FM in ordinary clinical practice [28].

The ability to identify indicators that are regularly linked to this illness would help clinicians diagnose FM more accurately, track the disease's progression, evaluate the effectiveness of treatment options, and perhaps create preventive programs [29]. There is evidence to suggest that peripheral inflammation and nerve damage are associated with miRNA expression in pain-related areas [30]. Because one of the primary symptoms of FM is chronic pain, understanding the relationship between the environment and genes related to pain may help understand the etiological mechanism underlying this disorder.

The IL-6 molecule through activating the Jak/STAT3 pathway plays an essential role in fibromyalgia pathogenesis by causing excessive chemokines production and triggering the activation of glial cells. This suggests that by controlling the IL-6 route, it may be possible to reduce pain-related symptoms in individuals suffering from fibromyalgia [31]. miRNAs can control the IL-6 expression. MiRNA-365, for instance, has been shown to down regulate the IL-6 production in HEK293 and HELA cells [32]. Our investigation revealed a significant difference in MicroRNA-217 between the patient and control groups, with the fold change indicating that fibromyalgia patients showed significantly lower levels of MicroRNA than the control group. Therefore, we speculate that miRNA-217 might be an upstream miRNA in fibromyalgia that controls IL-6.

MicroRNA-217 has a tight relationship with both fibrosis and inflammation. For instance, miR-217 role in inhibiting pancreatic ductal carcinoma was previously studied and was linked to targeting KRAS [33]. It was also related to the prevention of proximal tubule cell fibrosis by protecting dopamine receptor D2 [34]. Likewise, it was connected to the regulation of expression of silent information regulator 1 (SirT1), which is a key regulator of metabolic diseases and longevity whose expression is gradually decreased in several tissues with ageing [35]. Moreover, it influences glomerular mesangial cell fibrosis and the inflammatory response via the Sirt1/HIF-1a signaling pathway [14].

Our experiment's outcomes align with those of Jiongwei et al., who demonstrated that patients suffering from interstitial pneumonia had down-regulated miRNA 217 (*P* < 0.05). The double-luciferase reporter assay suggested that IL-6 was the target gene of miRNA-217. They hypothesize that the downregulation of miRNA-217 expression may facilitate interstitial pneumonia through IL-6, which might be related to the increase in the serum expression of IL-6 in patients with interstitial pneumonia [36].

Concerning miR-532, our analysis revealed that there was significant down-regulation in the fold change of miR-532 in fibromyalgia patients [median = 0.22, IQR: (0.11–0.42), *P* < 0.001] in comparison with controls (Table 3). A prospective explanation for this dysregulation could be through Tumor necrosis factor-α which serves as one of the most significant cytokines released by macrophages and microglia cells and demonstrates activity in the pathways of neural pain, through regulating pain signals and plays a critical role in the area of inflammation, where pain arises [37]. Furthermore, the body secretes substance P and corticotropin-releasing hormone while under stress. These two compounds both induce inflammation and facilitate the production of TNF-α [37]. It was found that compared to healthy people, FMS patients had increased amounts of TNF-α, substance P, and corticotropin-releasing hormone [38], It has been determined that the expression of miR-532-3p can antagonize the inflammatory disease conditions mediated by lipopolysaccharide LPS/TNFα by targeting the Apoptosis signal-regulating kinase 1 (ASK1)/p38 MAPK pathway [39].

Concerning the strength of our work, we aimed to be the first to investigate the possible association of sera levels of miR-217 and miR-532 in patients with fibromyalgia and correlate their expression levels to different clinical and biochemical disease criteria.

The current study has two limitations that need to be addressed to validate our results: (1) a limited sample size; and (2) a scientific functional publication that lacks sufficient evidence to demonstrate the molecular foundation or important functions of target genes in the pathophysiology of fibromyalgia. The exact functions played by these genes in the development of disease must be examined in larger-scale studies.

## Conclusion

According to our data, the fold changes of miR**-**217 and miR-532 in fibromyalgia patients were significantly lower than in controls, accordingly serum miR-217 and miR-532 could be used as potential biomarkers for diagnosis of fibromyalgia that may be used as therapeutic targets.

## Data Availability

No datasets were generated or analysed during the current study.
